# A metastatic invasive mole arising from iatrogenic uterus perforation

**DOI:** 10.1186/s12885-017-3904-2

**Published:** 2017-12-20

**Authors:** Yuanming Shen, Xiaoyun Wan, Xing Xie

**Affiliations:** 1 Department of Gynecologic Oncology, Women’s Hospital, School of Medicine, Zhejiang University, Hangzhou, 310006 China; 2 Women’s Reproductive Health Laboratory of Zhejiang Province, Women’s Hospital, School of Medicine, Zhejiang University, Hangzhou, China

**Keywords:** Mole pregnancy, Invasive mole, Uterine perforation

## Abstract

**Background:**

Invasive mole derives from hydatidiform mole, but its pathogenesis remains unknown. Invasive mole arising from iatrogenic uterine perforation has not been reported yet.

**Case presentation:**

A reproductive woman was admitted because she suffered form severe abdominal pain and acute intra-abdominal hemorrhage after suction evacuation due to misdiagnosis as inevitable abortion. The patient underwent hysteroscopy and laparoscopy, by which an iatrogenic uterine perforation and omentum and pelvic peritoneum metastases were confirmed. All lesions were removed and the final pathological diagnosis was metastatic invasive mole. The patient underwent post-operative chemotherapy with methotrexate and presented a good prognosis.

**Conclusion:**

Invasive mole arising form iatrogenic uterine perforation displays an unusual metastatic manner other than general invasive moles. The prevention of uterine perforation should be emphasized during suction evacuation for mole pregnancy.

## Background

Invasive mole is defined as the existence of edematous and/or degraded villus with trophoblastic proliferation in the myometrium or extra-uterine metastases which arises from myometrial invasion of hydatidiform mole via direct extension through tissue or venous channels. Previous studies have reported that malignant change occurs in approximately 15~20% of complete hydatidiform moles (CHMs) and less than 1~5% of partial hydatidiform moles (PHMs) [1–3]. Commonly, invasive mole is often clinically rather than histologically diagnosed based on persistent elevated serum human chorionic gonadotropin (hCG) level after the evacuation of mole tissues. The etiologic events contributing to the development of invasive mole are unclear except for some high-risk factors, such as regional differences or racial variations, older or younger maternal age, a lack of vitamin A in diet, and others [3, 4]. Invasive mole arising from iatrogenic event has not been reported yet.

Invasive mole generally limits in uterine myometrial invasion and extra-uterine metastases occur in only 5% of CHMs and rare cases in PHMs [3, 4]. Metastases are developed mainly through hematogenous spread and lung (80%) is the most common metastatic site, followed by vagina (30%), pelvis (20%), liver (10%), brain (10%), and others (<5%) [3–5]. Metastasis to the pelvic peritoneum or omentum, independent of uterine myometrial invasion and lung metastases, is extremely rare in invasive mole. Here, we reported a case of invasive mole that derived from iatrogenic uterine perforation and showed a special metastatic manner. This case awakes us that the prevention of uterine perforation should be emphasized during suction evacuation for mole pregnancy.

## Case presentation

**Fig. 1:**
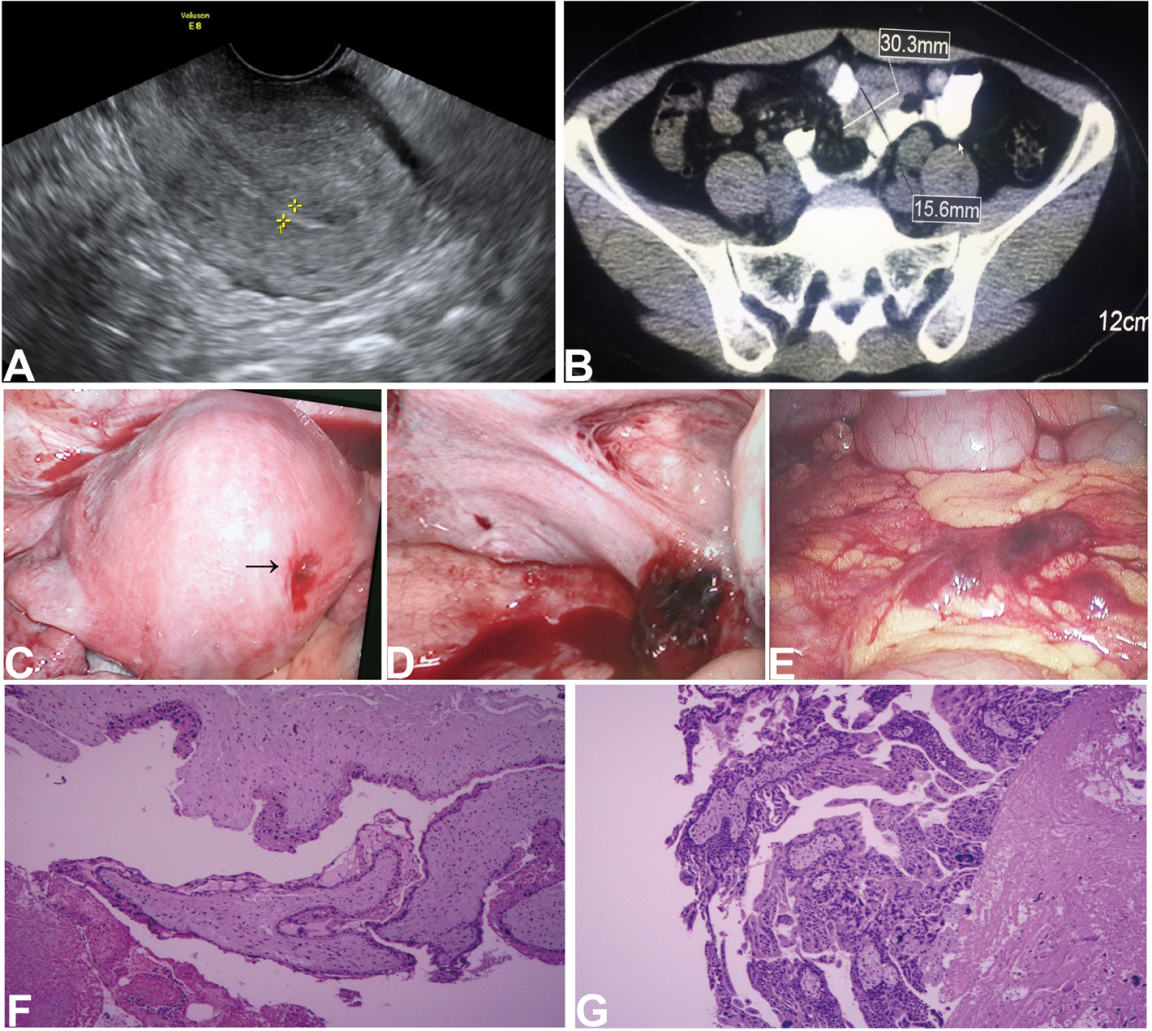
**a**-**b** An ultrasound examination (**a**) and a CT scan (**b**) of the uterine and pelvis. **c**-**e** An iatrogenic uterine perforation (arrow) (**c**) and the lesions [right uterosacral ligament (**d**) and omentum (**e**)] of mole under laparoscopy. **f**-**g** Edematous villus with trophoblastic proliferation was significant in omentum (**f**) and right uterosacral ligament (**g**)

A 34-year-old woman, G5P2A2, referred to our hospital (Women’s Hospital, School of Medicine, Zhejiang University, Hangzhou, China) because of irregular vaginal bleeding and elevated serum hCG level. She had undergone suction curettage one month ago due to initial diagnosis of inevitable abortion at approximately 8 weeks of gestation in a local hospital. Thenceforth, she suffered persistent vaginal bleeding with rebound serum hCG levels for 3 weeks. Weekly serum hCG levels were 5310 IU/L, 7600 IU/L, and 9121 IU/L after the second curettage, while the levels of other tumor markers were within the normal ranges. An ultrasound examination revealed normal ultrasonic echo in both uterine myometrium and parenchyma except for irregular echo of uterine endometrium and a little enlarged uterus (Fig. 1a). After admission to our hospital, a pathological diagnosis of CHM was confirmed according to the HE slices from first curettage. All the imaging data which were performed in local hospital were reanalyzed and reconfirmed. Additional abdomen and pelvis CT scan were done in our hospital. All the imaging scans were normal. Combining the symptoms, previous history of CHM, and elevated hCG level, the initial diagnosis was considered as gestational trophoblastic neoplasia (GTN). The patient suddenly presented severe abdominal pain in the second early morning after hospitalization and the ultrasound examination showed intra-abdominal hemorrhage.

Combined emergency hysteroscopy and laparoscopy were performed. Hysteroscopy revealed an empty uterine cavity without gestational tissue except for an old iatrogenic uterine perforation at right fundus of uterine. Under laparoscopy, an iatrogenic uterine perforation was confirmed. Totally 300 ml uncoagulated blood were collected in pelvic cavity and three mole lesions with dark-brownish, coin-sized bleeding spot were noted, one was at the pelvic peritoneum near the right uterosacral ligament (about 2 × 2 cm), another was at posterior uterine serosa (0.3 × 0.3 cm), and the third was on the omentum (3 × 2 cm) (Fig. 1c–e). Both sides of adnexae appeared normal. The iatrogenic uterine perforation was mended. All lesions of mole were removed. The abdomen and pelvis CT and MRI images were postoperatively re-reviewed, and a mass (3.03 × 1.56CM) on omentum was found in CT scan, as shown in Fig. 1b. The final pathological diagnosis of the three mole lesions was invasive mole. As showed in Fig. 1f and g, edematous villus with trophoblastic cells significantly invaded into the pelvic peritoneum and omentum metastatic sites. Thus, the patient was diagnosed as FIGO stage IV with score of 3 (1 for hCG level > 1000 U/L, 1 for >3 cm metastasis on omentum, and 1 for three identified metastases lesions). According to 2000 FIGO staging, a risk score of 6 and below is classified as low risk. Thus, the patient underwent chemotherapy with single methotrexate regimen (4 mg/kg at day 1–5, 2-week interval). The serum hCG level was rapidly decreased. At the end of the second course of methotrexate chemotherapy, the hCG level dropped to negative, and additional 2 courses of chemotherapy was given. The woman was followed up to one year and her hCG level was normal.

## Discussion and conclusions

Suction evacuation as the standard therapeutic strategy should be done as soon as possible after the diagnosis of hydatidiform mole [6]. Uterine perforation is a potential complication of uterine suction and surgical curettage, easily occurring in mole pregnancy because of a big and soft uterus [6]. The perforation may lead to the damage of intraperitoneal organs, even heavy intraperitoneal hemorrhage. This was a first reported case of invasive mole that resulted from iatrogenic uterine perforation, to the best of our knowledge. This patient was misdiagnosed as inevitable abortion before curettage. Imaginably, a sharp curettage was performed blindly which might lead to uterus perforation consequently. A proper surgical technique in evacuation of molar pregnancies is very important, including a sufficient preoperative evaluation to exclude hyperthyroidism, hypertension, severe anemia, and others; suitable dilation of cervical os according to gestational weeks; gently operation for dilation, suction, and curettage to avoid uterine perforation. Oxytocin infusion is recommended after cervix dilation during suction curettage. In general, the operation should be performed by an experienced gynecologist under ultrasound guidance.

Invasive moles have the same histopathological characteristics as that of a non-invasive hydatidiform mole except for the infiltration of the trophoblasts into the myometrium and the necrotic changes associated with it. Up to date, the pathogenesis of invasive mole still remains unknown. The dysfunction of oncogenes and anti-oncogenes might contribute to the malignant transformation of the trophoblasts in invasive mole, like in other malignancies [7]. For this case, we hypothesized that the trophoblasts might have had inherent genes dysfunction and uterine perforation only offered a metastatic path. Generally invasive mole causes invasion of villous fragments into the uterine myometrium, minority causes metastasis in distant organs (mostly to lung) via hematogenous metastasis [5]. Primary intra-abdominal involvement without uterine myometrial invasion and lung metastasis is extremely rare. However, this special case arising form iatrogenic uterus perforation represented an unusual metastatic manner other than general invasive mole. Metastases to peritoneum and omentum occurred independently of uterine myometrial invasion and lung metastases. We presumed that the trophoblasts might directly via the perforation site rather than venous channels, leading to peritoneum and omentum implantation. According to FIGO staging, this patient belonged to an advantage disease. It is unclear whether the prognoses are different between this patient and those with same stage through general metastatic manners. In our report, woman underwent four courses of single methotrexate chemotherapy after operation, the prognosis was good.

In summary, we reported a special case of invasive mole arising from iatrogenic uterine perforation, which showed an unusual metastatic manner other than general invasive mole. This avoidable case suggests that the prevention of uterine perforation should be emphasized during suction evacuation for mole pregnancy.

## Teaching points

1. A proper surgical technique in evacuation of molar pregnancy should be emphasized.
2. Invasive mole arising form iatrogenic uterine perforation displays an unusual metastatic manner.

## Abbreviations

CHMs: Complete hydatidiform moles
GTN: Gestational trophoblastic neoplasia
HCG: Human chorionic gonadotropin
MRI: Magnetic resonance imaging
PHMs: Partial hydatidiform moles

## References

[1] El-Helw LM, Hancock BW (2007). Treatment of metastatic gestational trophoblastic neoplasia. Lancet Oncol..

[2] Shih IM (2007). Gestational trophoblastic neoplasia—pathogenesis and potential therapeutic targets. Lancet Oncol.

[3] Seckl MJ, Sebire NJ, Berkowitz RS (2010). Gestational trophoblastic disease. Lancet.

[4] Brown J, Naumann RW, Seckl MJ, Schink J (2017). 15years of progress in gestational trophoblastic disease: scoring, standardization, and salvage. Gynecol Oncol.

[5] Seckl MJ, Sebire NJ, Fisher RA, Golfier F, Massuger L, Sessa C, ESMO Guidelines Working Group (2013). Gestational trophoblastic disease: ESMO Clinical Practice Guidelines for diagnosis, treatment and follow-up. Ann Oncol.

[6] Candelier JJ (2016). The hydatidiform mole. Cell Adhes Migr.

[7] Alifrangis C, Seckl MJ (2010). Genetics of gestational trophoblastic neoplasia: an update for the clinician. Future Oncol.

